# *Bifidobacterium animalis* subsp. *lactis* HN019 live probiotics and postbiotics: production strategies and bioactivity evaluation for potential therapeutic properties

**DOI:** 10.3389/fbioe.2024.1379574

**Published:** 2024-07-09

**Authors:** Sergio D’ambrosio, Azza Dabous, Saba Sadiq, Angela Casillo, Chiara Schiraldi, Elisabetta Cassese, Emiliano Bedini, Maria Michela Corsaro, Donatella Cimini

**Affiliations:** ^1^ Department of Environmental, Biological and Pharmaceutical Sciences and Technologies, University of Campania Luigi Vanvitelli, Caserta, Italy; ^2^ Department of Experimental Medicine, University of Campania “L.Vanvitelli”, Naples, Italy; ^3^ Department of Nutrition and Food Technology, An-Najah National University, Nablus, Palestine; ^4^ Department of Chemical Sciences, University of Naples “Federico II”, Complesso Universitario Monte S. Angelo, Naples, Italy

**Keywords:** *Bifidobacterium lactis*, probiotic, postbiotic, exopolysaccharide, parabiotic, zonulin

## Abstract

**Introduction:** *B. animalis* subsp. *lactis* HN019 is a commercially available well-characterized probiotic with documented effects on human health, such as the ability to enhance the immune function and to balance the intestinal microbiome. Therefore, optimizing the manufacturing process to improve sustainability, increasing biomass yields and viability, and avoiding animal -derived nutrients in the medium to meet vegan consumer’s needs, is currently of interest. Besides the established use of live probiotic cells, alternative supplements indicated as postbiotics, like non-viable cells and/or probiotics derived bioactive molecules might be considered as potential next generation biotherapeutics. In fact, advantages of postbiotics include fewer technological limitations, such as easier production processes and scale-up, and even higher specificity.

**Methods:** In this work, medium design together with different fermentation strategies such as batch, fed-batch and in situ product removal on lab-scale bioreactors were combined. Medium pretreatment by ultrafiltration and protease digestion was performed to reduce polysaccharidic contaminants and facilitate the purification of secreted exopolysaccharides (EPS). The latter were isolated from the fermentation broth and characterized through NMR, GC-MS and SEC-TDA analyses. The expression of TLR-4, NF-kb and IL-6 in LPS challenged differentiated CaCo-2 cells treated with EPS, live and heat-killed *B. lactis* cells/broth, was evaluated *in vitro* by western blotting and ELISA. Zonulin was also assessed by immunofluorescence assays.

**Results and Discussion:** The titer of viable *B. lactis* HN019 was increased up to 2.9 ± 0.1 x 10^10^ on an animal-free semidefined medium by applying an ISPR fermentation strategy. Medium pre-treatment and a simple downstream procedure enriched the representativity of the EPS recovered (87%), the composition of which revealed the presence of mannuronic acid among other sugars typically present in polysaccharides produced by bifidobacteria. The isolated EPS, live cells and whole heat inactivated broth were compared for the first up to date for their immunomodulatory and anti-inflammatory properties and for their ability to promote intestinal barrier integrity. Interestingly, EPS and live cells samples demonstrated immune-stimulating properties by downregulating the expression of TLR-4 and NF-kb, and the ability to promote restoring the integrity of the intestinal barrier by up-regulating the expression of zonulin, one of the tight junctions forming proteins. Postbiotics in the form of heat killed broth only reduced NF-kb expression, whereas they did not seem effective in the other tested conditions.

## 1 Introduction

*Bifidobacterium* is a genus of Gram-positive, often branched anaerobic bacteria. Various bifidobacteria strains have been widely used commercially and are usually available on the market as functional components of dairy-based probiotic drinks (e.g., *Bifidobacterium breve* strain Yakult, Yakult Japan, *Bifidobacterium (animalis) lactis*, Danone France, *etc.*) (Felis and Dellaglio, 2007). This genus includes bacterial species of most prevalent groups of culturable anaerobic bacteria within the human and animal gastrointestinal tract (GIT), and among the first to colonize the human GIT (Velraeds et al., 2000). The ability of bifidobacteria to exert different health-promoting effects in their host is widely claimed; there is evidence showing that the presence of these microorganisms improves gut homeostasis and functionality (Tojo et al., 2014; Rivière et al., 2016). Moreover, they were demonstrated to provide protection against pathogen proliferation, and stimulation of the immune system (Ruiz et al., 2017; Alessandri et al., 2019). *B. lactis* Bi-07 was shown to improve the phagocytic activity of monocytes and granulocytes in healthy elderly adults after 4 weeks’ administration (Maneerat et al., 2013). Furthermore, a recent study demonstrated the antibacterial activity of *B. breve* YH68 against *Clostridioides difficile* involving the inhibition of growth, production of spores and toxins, and expression of virulence genes (Yang and Yang, 2019).

At the same time, bifidobacteria produce different metabolites, such as vitamins, polyphenols, short-chain fatty acids (SCFAs) that are believed to be beneficial for both epithelial host cells and gut microorganisms (Bottacini et al., 2014; Rivière et al., 2016). In particular, the commercial strain *B. animalis* subs. *lactis* HN019 isolated from yogurt and marketed as HOWARU Bifido by Danisco (United States) was studied for a variety of important traits and for its probiotic functions. In particular, first studies regarded its role in the prevention and treatment of diarrhea (Shu et al., 2001; Thibault et al., 2004). It was also observed that this strain establishes a protective microflora in human subjects (Gopal et al., 2003). Moreover, different studies focused on its possible role as immunostimulant in elderly people, that would improve both natural and acquired immunity (Arunachalam et al., 2000; Chiang et al., 2000). Besides the interest towards live probiotics, attention focused on postbiotics in recent years (Ciernikova et al., 2023; Vinderola et al., 2023; Sepordeh et al., 2024), “Inanimate microorganisms and/or their components that confer a health benefit on the host” as defined in 2021 by the “The International Scientific Association of Probiotics and Prebiotics” (ISAPP) (Salminen et al., 2021). The latter are studied as Next-Gen biotherapeutics to provide gut health through antibacterial, antioxidant, and anti-inflammatory activities (Tong et al., 2023). In a Caco-2 *in vitro* model, heat-killed *Limosilactobacillus reuteri*, for instance, showed anti-adhesion ability against various enteropathogens (Singh et al., 2017). Heat-inactivated *B. animalis* subs *lactis* CECT8145 improved obesity and metabolic disorders in cafeteria-diet fed rats and, interestingly, it also reduced fat deposits in norm weight animals (Caimari et al., 2017).On the other hand, a recent study focused on the possible action of different postbiotics from *Lacticaseibacillus paracasei, Latilactobacillus sakei* and *Lacticaseibacillus rhamnosus* as treatment for pediatric atopic dermatitis resulting in significant benefits in children affected by this disorder (Sajo et al., 2023).

Therefore, this study was designed to develop high cell density production of *B*. *animalis* subsp. *lactis* HN019 in bioreactor up to the 22 L scale, to be used as source of live cells, EPS and other metabolites secreted in the fermentation medium. *B. lactis* strains, as a matter of fact, produce bacteriocins and exopolysaccharides (EPSs) during the early fermentation process that are interesting from an applicative point of view due to numerous potential biological activities (Lee and O’Sullivan, 2010; Oerlemans et al., 2021). EPSs can stimulate the growth of other probiotic strains, inhibit pathogen bacterial adhesion to the intestinal epithelium, increase intestinal barrier integrity by up-regulating the expression of tight junction forming proteins, and influence the immune system acting directly or indirectly on Toll-like receptors (Oerlemans et al., 2021). In this study, live *B. lactis* HN019 (probiotics), purified EPSs and whole broth containing heat-inactivated/killed cells (postbiotics) were compared to evaluate their potential health benefits.

## 2 Materials and methods

### 2.1 Bacterial strain and medium

*B. animalis* subsp. *lactis* HN019 was provided by “Centro Sperimentale del latte S.r.l.-Sacco System” in the framework of the MISE project “INCUBE” and stored at −80°C in Mann, Rogosa and Sharpe (MRS) broth. Twenty % (v/v) glycerol stock suspensions prepared with exponentially growing cells were stored at − 80°C.

All medium components and salts were supplied by Sigma-Aldrich (St. Louis, MO, United States). Yeast extract was furnished by Organotechnie (La Corneuve, France), while sulphuric acid was purchased by Biochem s.r.l. (Turin, Italy). The different semi-defined media used for growth experiments are listed in Table 1. All the carbon sources to a final concentration of about 30 g/L, were filter sterilized (0.22 μm) and then added to all semi-defined media after autoclaving.

**TABLE 1:** Composition of the semi-defined media used in 100 mL bottle experiments.

Component (g/L)	Medium 1	Medium 2	Medium 3	Medium 4	MRS
Yeast extract	5	15	5	20	5
Soy Peptone	20	15	20	15	—
Casein peptone, tryptic digest	—	—	—	—	10
Meat extract	—	—	—	—	10
K_2_HPO_4_	—	1	2	—	2
KH_2_PO_4_	—	1	—	—	—
Na_2_HPO_4_	—	—	—	5	—
NaH_2_PO_4_	2	—	—	10	—
MgSO₄ * 7 H₂O	0.1	0.2	0.1	—	0.2
MnSO₄ *H₂O	0.05	0.01	0.05	0.05	0.05
Tween80 (mL/L)	1	1	2	1	1
C_6_H_8_O_7_ 2NH_3_	2	—	2	2	2
Sodium acetate	5	—	5	—	5
MgCl_2_ * 6 H_2_O	—	—	—	—	—
NaCl	—	0.01	—	—	—
FeSO_4_	—	0.01	—	0.1	—
Lithium sulphate	—	—	2	—	—
Maleic acid	—	—	—	0.1	—
L-Cysteine	2	0.5	2	0.5	—

### 2.2 Bottle experiments

Seed cultures were prepared under sterile conditions by adding the stock solution (about 10 OD_600_) of *B. lactis* HN019 into 0.1 L of medium in a 0.1 L pyrex screw-cap bottle to reach a starting concentration of 0.1 OD_600_. The bottle was incubated in a rotary air shaker incubator (model Minitron, Infors, Bottmingen, Switzerland) at 37°C and 150 rpm. Small scale experiments lasted 24 h. Samples were withdrawn from the culture for glucose consumption and acid production analysis. All experiments were conducted at least in triplicate.

### 2.3 *In situ* product removal (ISPR) processes by microfiltration

A polypropylene membrane, Accurel PP, consisting of a long capillary (5–10 m) with an inner diameter of 0.2 mm and a cutoff of 0.22 µm was used for the construction of the microfiltration module. Briefly, 15 capillaries 15- cm long were obtained from the original membrane and assembled with a silicon adhesive; one side was closed and the other was sealed to a connector. The module was connected to a peristaltic pump (model 313 U; Watson Marlow, Falmouth, United Kingdom) equipped with a silicon tube, with an inner diameter ϕ = 2 mm, which provided the driving force for transmembrane flux. Considering the geometry of the module, the total filtering area measured 600 cm^2^. Before using the module, the membranes were treated with a 70% ethanol solution for 1 h to improve hydrophilicity. The microfiltration module was placed inside the fermentation vessel and fixed vertically to a baffle to have a high turbulence near the filtering surface thereby minimizing fouling. Finally, the membrane modules were *in situ* sterilized together with the medium at 90°C for 45 min to avoid deconstructing the adhesive silicone.

### 2.4 Fermentation processes

**FIGURE 1:**
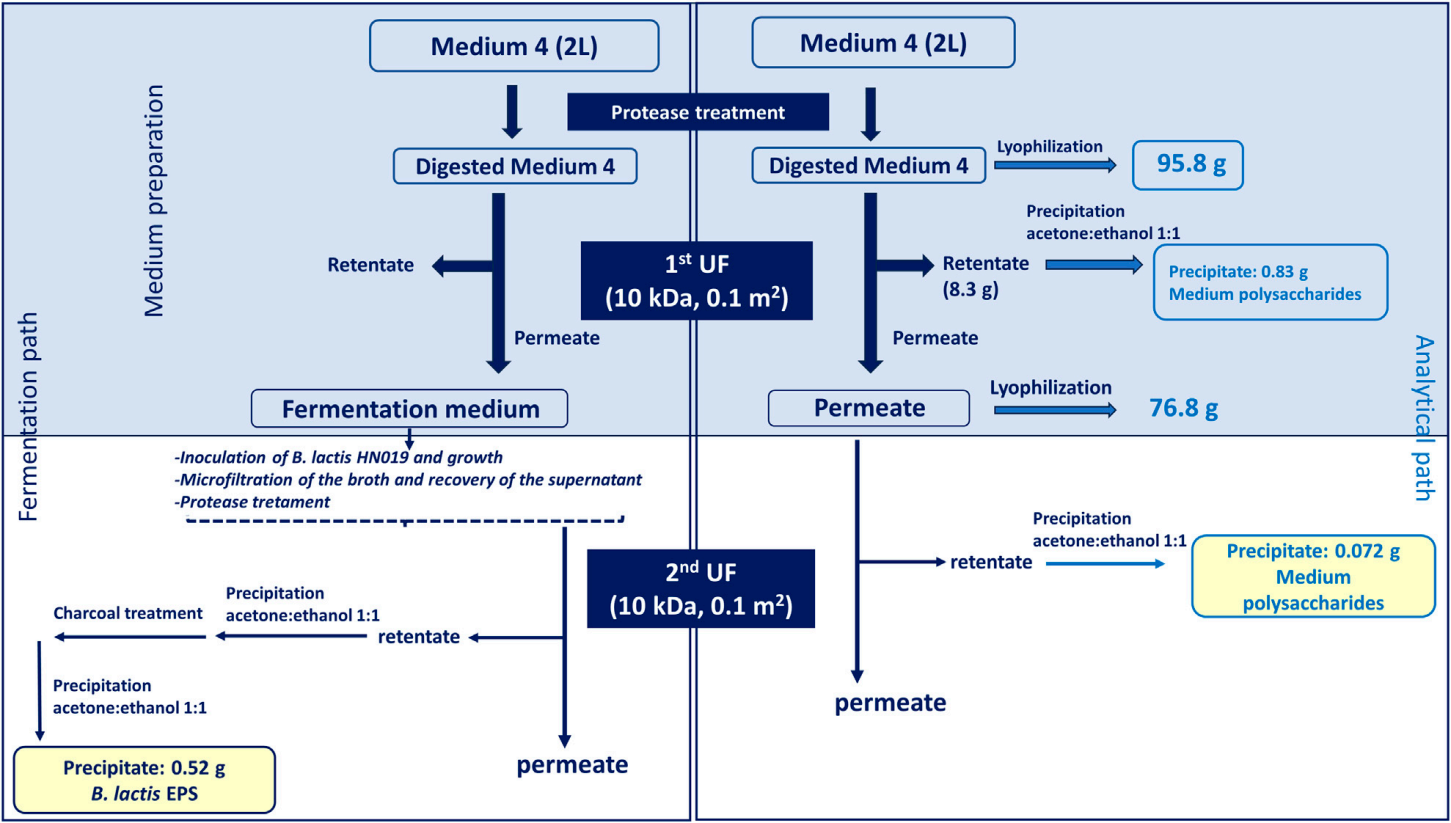
Downstream process applied on the fresh medium, to eliminate potential contaminating polysaccharides present in complex nitrogen sources, and on the fermentation broth to recover the EPS produced by *B. lactis* HN019. The top light blu box indicates that the treatment applied to the medium for analytical purposes (right) and for fermentation experiments (left) is common up to that point for both strategies. The analytical path served in the first part (light blu box) to quantify the polysaccharides present in the medium thereby establishing the amount of eliminated contaminants; in the second part (white box) it helped estimating the percentage of polysaccharide that could still contaminate the EPS sample in the worst-case scenario (no medium consumption).

Fermentation experiments were performed in a Biostat CT plus and on Biostat C (Sartorius Stedim, Gottingen, Germany) with a working volume of about 2.2 and 18 L, respectively. Temperature was set to 37°C, pH was controlled at 6.2 and agitation was fixed at 150 rpm. A 25% (v/v) hydroxide ammonium solution was used as base while H_2_SO_4_ 30% (v/v) as acid for pH maintenance. Batch experiments were conducted on the semi-defined medium described in the previous paragraph with glucose as carbon source. Before each experiment, 1 mL of a concentrated stock solution of about 50 OD_600_ was inoculated in 0.45 L of medium and incubated at 37°C and 150 rpm for 8 h. A peristaltic pump (model 313 U, Watson-Marlow, England) was used to transfer the pre-culture inside the vessel to reach 10% (v/v) of the working volume inside the fermenter. Experiments were performed without air insufflation in the fermenter. Experiments lasted from 16 to 30 h. Each fermentation experiment was performed at least in triplicate. Batch experiments specifically aimed at purifying the EPSs were performed on a processed semi-defined medium. After preparation according to the recipe described above, the medium was treated with 20 U/L protease from *Aspergillus oryzae* (Sigma-Aldrich, Missouri, United States) at room temperature for 1 h and next ultrafiltered on 10 kDa polyethersulfone membranes (GE Healthcare, Illinois, United States) with 0.1 m^2^ of filtration area. Tangential flow filtration was performed on a Sartoflow alpha (Sartorius Stedim, Gottingen, Germany) system connected with a thermostatic bath that kept a constant temperature of about 20°C–25°C. The retentate was concentrated to about 1/10th of the initial volume, washed with 3 volumes of milliQ water and precipitated with 2 volumes of a 1:1 (v/v) ethanol:acetone solution, after adjusting the conductivity to 20 mS/cm, and dried in a vacuum oven at 40°C over-night to quantify medium polysaccharides (Figure 1, analytical path).

### 2.5 Fed-batch experiments

The concentrated feeding solution contained 200 g/L glucose and 50 g/L of both yeast extract and soy peptone. After the batch phase, a constant feeding addition rate of about 7 g/L· h (of carbon source) was used for all experiments for up to 30 h.

### 2.6 ISPR experiments

At the end of the batch phase when the residual glucose concentration in the bioreactor was equal to about 1 g/L, the microfiltration mode was activated. Feeding was performed as described for the fed-batch experiments. The working volume was kept constant by a level sensor, controlling the addition of salt solution, when necessary, prepared according to the medium recipe. Frequent (about every hour) 2 min back-flushings with normal saline solution were performed to maintain a flux that was on average 75% of the set point.

Dry cell weight was evaluated by centrifuging 50 mL of broth in a falcon tube at 6500 × g at 4°C for 30 min. After discarding the supernatant, the pellet was washed with 0.5 volumes of normal saline solution and then transferred in a pre-weighted Eppendorf tube. The pellet was dried overnight (o/n) at 70°C before measuring the dry weight. Viability was evaluated by serially diluting the samples and plating on MRS-agar medium. Plates were incubated at 37°C for 36 h before counting viable cells. Each sample was analyzed in triplicate.

### 2.7 Spray drying

The broth from 22 L fermentations was concentrated by microfiltration on a 0.22 μm hollow fiber module. Sixteen liters of culture were filtered on 0.98 m^2^ (with 1250 PES fibers, 1 mm thick) to separate biomass and supernatant for the recovery of the biomass. The concentrated biomass (about 2.9 L) was diafiltered with 2 volumes (8.5 L) of sterile phosphate buffer solution (PBS) and after addition of trehalose and sucrose (ratio 1:1) it was spray-dried on a Mobile minor™ (Gea Process Engineering, Düsseldorf, Germany). Based on the theoretical wet to dry biomass ratio, a 1:1 ratio between dry biomass and total sugars was maintained. The sample was fed into the spray dryer at 2.2 L/min, with a peristaltic pump (model 730U, Watson-Marlow, England). The inlet and outlet temperatures were set to 165°C and 85°C, respectively, while the atomizer pressure was set to 1 bar.

### 2.8 Preparation of heat inactivated *B. lactis* HN019 broth

The broth from batch fermentations on the 3 L scale bioreactor was used to produce heat inactivated postbiotics. Briefly, cells were heat-inactivated at the end of the fermentation process, at 90°C for 1 h with 150 rpm agitation. The heat-treated broth was collected in sterile conditions and transferred to a 2 L glass balloon and frozen at −80°C for 3 h. Finally, freeze-drying was performed in a bench scale freeze dryer (Beta 2- 8 LSC plus, Christ, Gefriertrocknungsanlagen, Germany) by setting sublimation at −90°C at a chamber pressure of 1 mbar for 18 h, followed by a second drying lasting 2 h at 0.01 mbar.

### 2.9 HPLC quantification of sugars, organic acids and ethanol

Broth samples of about 10 mL were withdrawn during the experiments to run HPLC analyses of substrate consumed, and acid produced. The broth was centrifuged for 15 min at 6500 × g to separate biomass and recover the supernatant. One mL was then ultrafiltered and diafiltered on 3 kDa centrifugal filter devices (Centricon, Amicon, Sigma-Aldrich) at 12,000 × g and the permeate was analysed for the determination of the concentrations of fructose, glucose, lactic and acetic acids, and ethanol by HPLC (UHPLC Dionex Ultimate 3000; Thermofisher) on a Alltech IOA-2000 column (500 mm × 6.5 mm ID) as previously reported (D’ambrosio et al., 2022).

### 2.10 Purification of exopolysaccharide

The EPS was recovered from the supernatant obtained by microfiltration at the end of the fermentation process. The supernatant was treated with 20 U/L protease from *A. oryzae* (Sigma-Aldrich, Missouri, United States) at room temperature for 1 h and next ultrafiltered on 10 kDa polyethersulfone membranes (GE Healthcare, Illinois, United States) with 0.1 m^2^ of filtration area. Tangential flow filtration was performed on a Sartoflow alpha (Sartorius Stedim, Gottingen, Germany) system connected with a thermostatic bath that kept a constant temperature of about 20°C–25°C. The retentate was concentrated to about 1/10th of the initial volume and washed with 3 volumes of milliQ water to remove salts and low molecular weight molecules still attached to the membrane. The retentate from the ultrafiltration step was then precipitated with 2 volumes of a 1:1 (v/v) ethanol:acetone solution, after adjusting the conductivity to 20 mS/cm, and dried in a vacuum oven at 40°C over-night. As indicated in Figure 1 the same treatment was applied to the unused medium up to this point to differentiate medium polysaccharides from produced EPSs and quantify them. The obtained powder was then suspended at a concentration of 20 g/L in milliQ water and treated with 1% (w/v) of activated charcoal (Supelco Analytical, Sigma-Aldrich, Missouri, United States) in batch for 1 h at room temperature. The suspension was filtered on Velapad 60 filtration system (PALL Corporation, Milan, Italy) with nitrocellulose filters (BECO—PR Steril S80) purchased from Fluxa Filtri (Milan, Italy). The solution was precipitated with 2 volumes of a 1:1 (v/v) ethanol:acetone solution, and the pellet was dried as described above.

### 2.11 Phenol-sulfuric acid assay for exopolysasccharide quantification

Quantification of polysaccharides was performed by phenol-sulfuric acid test (DuBois et al., 1956), a colorimetric assay used for the determination of total sugars. During hydrolysis pentoses are dehydrated to furfural, and hexoses to hydroxymethylfurfural producing a yellow-gold color in the presence of phenol. The calibration curve was obtained with standard solutions of D (+) glucose at concentrations ranging from 0.01 to 0.1 mg/mL. Briefly, 200 μL of standards/sample were placed in a reaction tube with 200 μL of aqueous solution of phenol 5% w/v. Then, 1 mL of concentrated sulfuric acid (98% w/w) was added, and the reaction tube was quickly closed and after vigorous shaking, the reaction was carried out for 30 min at 30°C. Sample absorbance was read at 490 nm. The blank was obtained by adding water to the reaction mixture.

### 2.12 Characterization of the exopolysaccharide by SEC-TDA

SEC–TDA 305 (Malvern, Milan, Italy) was used for the analyses of EPS. The instrument is equipped with a triple detector array module including a refractive 11 index detector (RI), a four-bridge viscosimeter (VIS), and a laser detector (LS) made of a right-angle light scattering 12 (RALS) detector and a low-angle light scattering (LALS). The OmniSEC software program was used for the acquisition and analysis of the data. Two TSK–GEL GMPWXL columns (Tosoh Bioscience, Tokyo, Japan Cat. No. 8-08025, hydroxylated polymethacrylate base material, 100–1000 Å pore size, 13 μm mean particle size, 7.8 × 30.0 cm) in series, preceded by a TSK–GEL guard column GMPWXL (Tosoh Bioscience, Cat. No. 08033, 12 μm mean particle size, 6.0 × 4.0 cm) were used. An isocratic elution with 0.1 M NaNO_3_ aqueous solution (pH 7.0) at a flow rate of 0.6 mL/min was carried out. Analyses were performed at 40°C with a running time of 50′. The dn/dc used was equal to 0.150 mL/g. Universal calibration for the determination of K1, K2, and K3 was performed by using a polyethylene oxide (PEO) standard (22 kDa PolyCAL, Viscotek).

### 2.13 GC-MS

Monosaccharides were detected by Gas Chromatography-Mass Spectrometry after derivatization as acetylated methyl glycosides (AMGs) as already reported (Casillo et al., 2015). Briefly, 1 mg of the sample was treated with hydrogen chloride—methanol solution −1.25 M HCl for 16 h, at 80°C. Then, the sample was dried and acetylated with Ac_2_O and pyridine at 100°C for 30 min. The mixture was dissolved in chloroform and extracted three times with water. The organic phase was dried, dissolved in acetone and injected into GC-MS. Acetylated methyl glycosides were analyzed on an Agilent Technologies gas chromatograph 7820A equipped with a mass selective detector 5977B and an HP-5 capillary column (Agilent, 30 m × 0.25 mm i.d.; flow rate, 1 mL min^−1^, He as carrier gas), by using the following temperature program: 140°C for 3 min, then 140 → 240°C at 3°C min^−1^.

### 2.14 Analysis of exopolysaccharide by NMR

^1^H-NMR spectrum of EPS was measured at 25°C on a 10 mg/mL solution in D_2_O using a Bruker Avance-III 600 MHz instrument (Billerica, MA, United States) equipped with a cryo-probe. A 0–8 ppm spectral window, 16 scans, and 65,536 data points for each scan were set as parameters under a digital quadrature detection (DQD) spectrum acquisition mode. Data were processed using the data analysis packages integrated with Bruker TopSpin™ 4.0.5 software.

### 2.15 Cell culture and treatment conditions

To investigate the effect of live *B. lactic* (probiotics), heat-killed *B. lactis* broth (postbiotics), and different concentrations of EPS (1.5, 0.5 and 0.1 mg/mL) on intestinal cell permeability and on the inflammatory cascade, Caco-2 (Human Caucasian colon adenocarcinoma ATCC HTB 37™) cells were used, since they can differentiate on a specific medium within 3 weeks cultivation and mimic normal intestinal enterocytes in villi formation and tight junctions.

Specifically, Caco-2 were cultivated at 37°C and 5% CO_2_ and fully differentiated in Dulbecco`s modified eagle medium (DMEM) containing glucose and glutamine and supplemented with 10% fetal bovine serum (FBS), 1% Penstrep. The cells were grown in a sterile 25 cm^2^ flask at a concentration of 3 × 10^5^ to confluence for 21 days by changing the medium every 2 days (Fusco et al., 2021).

To disrupt intestinal barrier damage and induce inflammation, Caco-2 cells were seeded in 12 wells plates at a density of 1 × 10^5^ cells/well. The cells were then stimulated with 20 µg/mL LPS from *Salmonella* minnesota (Enzo Life Sciences, Farmingdale, NY, United States). After 24 h, supernatants were replaced with the following treatments each in triplicate: 1 × 10^7^ CFU/mL live *B. lactis* HN019 (1:100 Caco-2:CFU/mL *B. lactis* HN019) (di Vito et al., 2022), heat-killed *B lactis* HN019 1 × 10^7^ in the broth, and *B. lactis*-derived EPS were added in three different concentrations, namely, 1.5, 0.5, and 0.1 mg/mL. Three wells remained without LPS treatment as control.

### 2.16 Western blotting analysis for NF-kß, TLR-4

The proteins were isolated from differentiated Caco-2 cells after being treated as previously described. Cells were detached by trypsin, cell pellets were homogenized with RIPA buffer (Cell signaling technology) in cold ice for 30 min, and the total extract was cleared by centrifugation for 30 min and assayed for protein content by Bradford`s protein quantification method. About 50 µg/mL of proteins from each lysate were separated by 10% sodium dodecyl-polyacrylamide gel electrophoresis (SDS-PAGE) and transferred to nitrocellulose membrane. To verify equal loading and transfer efficiency, membranes were stained with 10% Ponceau S stain for 2 min.

Before adding the primary antibody, the membrane was incubated with 5% w/v skimmed milk dissolved in Tris-buffered saline and 0.05% v/v Tween (TTBS) for 15 min, to avoid any non-specific binding. Anti-TLR-4 (Abcam, Cambridge, United Kingdom) and anti NF-kß (Santa Cruz, Dallas, TX, United States) were diluted 1:500 in bovine serum albumin (BSA) and incubated at 4°C overnight. The membrane was then washed three times with TTBS and incubated with horseradish peroxidase-conjugated anti-mouse and anti-rabbit secondary antibodies for 2 h at room temperature. The membrane was washed again with TTBS, and the blots were developed by ECL system (Elabscience, Huston, TX, United States). GAPDH (Santa Cruz Biotechnology, Dallas, TX, United States, diluted 1:1000) was used as gel loading control. Finally, signal density was measured using ImageJ software, and each value was normalized to the value of GAPDH. Experiments were performed in triplicate.

### 2.17 Immunofluorescence analysis of tight junction protein zonulin (ZO-1)

To investigate the ability of live *B. lactis*, heat-killed *B. lactis*, and EPS to preserve the integrity of intestinal cell protein Zonulin (ZO-1), differentiated Caco-2 cells were seeded in well chambers at a density of 5 × 10^4^ cells/well, and they were grown for 48 h to reach 80% confluence. The cells were insulted with LPS (20 µg/mL) from *Salmonella minnesota* and after 24 h the samples (live *B. lactis*, heat-killed *B. lactis*, and EPS) were added and incubated for another 24 h. The supernatants were then discarded and the cells were washed with BPS, fixed with 4% (w/v) paraformaldehyde, and permeabilized with a solution of Triton X-100 at 0.2% (v/v) in PBS. The primary antibody against ZO-1 (Abcam, Cambridge, United Kingdom) was diluted 1:100 and incubated in each well overnight at 4°C. Subsequently, the slices were incubated with a FITC-conjugated goat anti-rabbit secondary antibody (ThermoFisher Scientific, United States) (diluted 1:100) for 1 h at room temperature and coated using the mounting medium with aqueous-DAPI. Fluorescence intensity was measured by ImageJ software and the intensity of zonulin was normalized to the intensity of the dapi.

### 2.18 Measurement of pro-inflammatory cytokine IL-6 using ELISA assay

As previously described, differentiated Caco-2 cells were stimulated with 20 µg/mL LPS, after 24 h of incubation the supernatants were replaced with inactivated DMEM medium containing the previously mentioned treatments (live cells, heat killed cells and EPS (1.5, 0.5, and 0.1 mg/mL) from *B. lactis* HN019. At the end of the experiment, the supernatants were collected by centrifugation of the cell culture medium at 3,000 × g for 10 min. The secretion levels of IL-6 were measured using a human IL-6 ELISA kit (Mabtech, AB, Sweden) according to the manufacturer’s instructions. All samples were assayed in triplicate. The absorbance was read at 450 nm using GloMax^®^ Discover microplate reader.

## 3 Results

### 3.1 Bottle experiments

**FIGURE 2:**
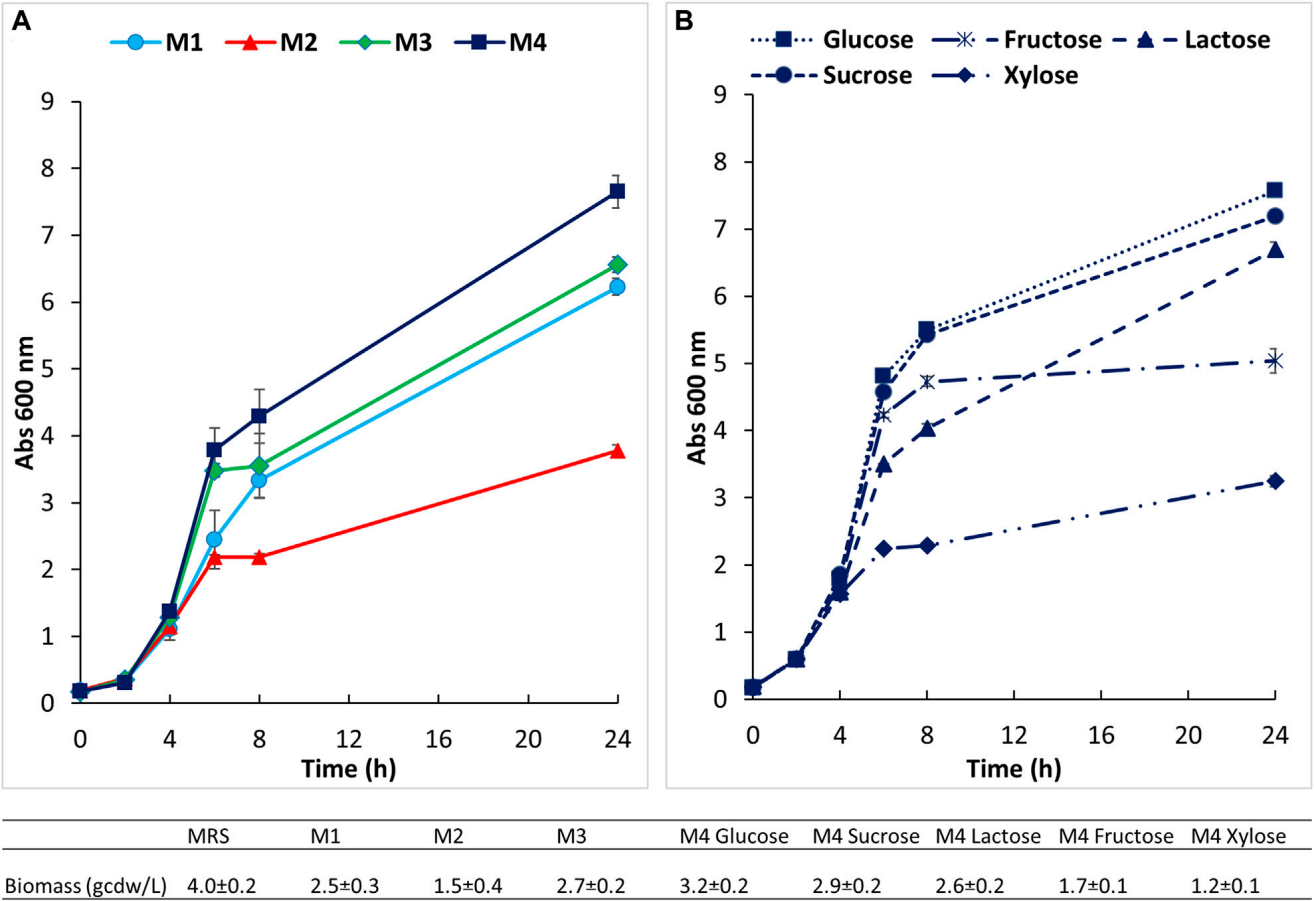
Small scale preliminary growth experiments conducted in 100 mL bottles at 37°C and 150 rpm. **(A)** Growth of *B. lactis* HN019 on four semi defined media; **(B)** growth on medium 4 supplemented with different carbon sources.

The main carbon and complex nitrogen sources, as well as salts, surfactant (tween) and other components were assayed in preliminary small scale screening experiments to define the most appropriate medium for the growth of *B. lactis* HN019, in comparison to MRS. The results of biomass production, showed as absorbance at 600 nm, on four tested media are indicated in Figure 2. As expected a faster growth and a final higher titer of biomass (OD_600_ 9.5 ± 1.0, viability 3.3 ± 1.1 × 10^9^) were achieved on the MRS medium that is extremely rich of complex and animal derived nitrogen sources. Among the simplified media (M1 to M4), a longer exponential phase, accompanied by a significantly higher final titer of biomass equal to 7.7 ± 0.2 OD_600_ and 3.2 ± 0.2 g/L of dry cell weight (Figure 2A), were observed on the medium that contained the highest total amount of yeast extract and soy peptone, medium 4. The concentration of viable cells obtained on this medium was equal to 2.3 ± 0.4 × 10^9^.

Growth of the strain under study was next evaluated on this medium (medium 4) supplemented with different carbon sources at the same initial concentration of about 30 g/L (Figure 2B). *B. lactis* HN019 reached very similar final OD and cell dry weight (3.3 ± 0.2 and 2.9 ± 0.1 g/L) on media containing sucrose and glucose, therefore, glucose was selected to assess growth in bioreactor experiments. Growth inhibition caused by lactic acid (LA) was also studied in bottle experiments on medium 4 supplemented with 15 and 30 g/L of LA. Biomass production was followed during first 7 h and the specific growth rate (μ) was calculated as linear regression of OD measurements. The μmax determined experimentally in the control medium was equal to 0.42 h^−1^ whereas a decrease to 0.34 and 0.20 h^−1^ was found in the presence of 15 g/L and 30 g/L of LA, respectively.

Further small-scale experiments were performed to evaluate the effect of the addition, or, higher concentration of three components, namely, L-cysteine, maleic acid and lithium sulfate to the previously selected medium 4. Results reported in Table 2 indicate, as highlighted by classic Anova and *post hoc* corrections, that either the addition of lithium sulfate or a higher concentration of maleic acid slightly improve biomass titers.

**TABLE 2:** Small scale growth experiments performed in 100 mL bottles at 37°C and 150 rpm on medium 4. Experiments were performed in duplicate. = indicates that the T medium contains the same concentration of component of that present in the control medium (M4). *Indicates that *p* < 0.05 in classic Anova with *post hoc* corrections, compared to the result obtained on M4.

Component (g/L)	CTR (M4)	T1	T2	T3	T4
Yeast extract	20	=	=	=	=
Soy peptone	10	=	=	=	=
Sodium phosphate monobasic	10	=	=	=	=
Sodium phosphate dibasic	5	=	=	=	=
Ammonium citrate dibasic	2	=	=	=	=
Tween 80	1	=	=	=	=
L-Cysteine	0.5	2	=	=	2
Iron sulphate	0.1	=	=	=	=
Maleic acid	0.1	=	0.5	=	0.5
Manganese sulfate	0.05	=	=	=	=
Lithium Sulfate (Li_2_SO_4_)	-	-	-	2	2
Biomass (Abs 600 nm)	7.77 ± 0.01	7.59 ± 0.04	8.27 ± 0.01*	8.20 ± 0.14*	7.68 ± 0.08

### 3.2 Fermentation experiments

Growth of *B. lactis* HN019 was assessed in a bioreactor on medium 4 under controlled and fixed conditions of pH, temperature, and aeration rate. Results of all fermentation experiments are summarized in Table 3.

**TABLE 3:** Fermentation experiments performed on Biostat CT plus (3L) ISPR, *In-situ* product removal; LA, lactic acid. Experiments were performed at least in triplicate. Data were analyzed by two-tailed non homoscedastic Student’s t-test. * indicates *p* < 0.05 ISRP vs. Batch; ** indicates *p* < 0.005 ISRP vs. Batch; # indicates *p* < 0.05 ISRP vs. Fed-Batch; ## indicates *p* < 0.005 ISRP vs. Fed-Batch; £ indicates *p* < 0.05 Fed-Batch vs. Batch; ££ indicates *p* < 0.005 Fed-Batch vs. Batch.

Fermentation strategy	Viability (CFU/mL)	OD_600_	Glucose consumption (g/L)	Lactic acid (g/L)	Y_LA/s_ (g/g)	Y_x_/_s_ (g/g)	Y_LA_/_x_ (g/g)	r_LA_ (g/L*h)
*Batch*	4.9 ± 2 × 10^9^	18.6 ± 3.4	28.6 ± 4.9	26.0 ± 2.0	0.9 ± 0.08	0.20 ± 0.04	4.1 ± 0.5	2.1 ± 0.6
*Fed-Batch*	1.5 ± 0.3 × 10^10 ££^	25.0 ± 1.0^££^	70.3 ± 13.5^£^	82.9 ± 14.8^£^	1.2 ± 0.06^£^	0.25 ± 0.02	7.9 ± 0.6	3.2 ± 0.4^££^
*ISPR*	2.9 ± 0.1 × 10^10 ##,**^	39.7 ± 3.5 ^##,**^	38.2 ± 4.2^##^	31.2 ± 1.8^##^	0.9 ± 0.22^##^	0.34 ± 0.10^#^	2.6 ± 0.2^##, *^	1.0 ± 0.1^##, *^

The growth rate, glucose consumption and LA production were evaluated during the processes. Short batch experiments with 30 g/L of initial glucose demonstrated a viability of about 4.9 × 10^9^ ± 2.0 CFU/mL after 10 h of growth. This value decreased to 0.9 × 10^9^ CFU/mL after 24 h (data not shown).

A higher final viability was obtained by using FB and ISPR strategies, the latter, in particular, increased the final concentration of viable cells by 60 fold, compared to results obtained in batch experiments, reaching about 2.9 × 10^10^ ± 0.2 CFU/mL and 39.7 ± 3.5 OD_600_. ISPR fermentations showed the highest Y_x/s_ equal to 0.34 ± 0.1 g/g, while production of LA was prompted in fed-batch experiments in which a Y_LA/s_ of 1.2 ± 0.1 g/g and a final concentration of LA of 82.9 ± 14.8 (Tab. 3) were obtained. No residual glucose was found in all types of fermentation experiments.

### 3.3 Purification and formulation of probiotic biomass

About 17 L of the broth were microfiltered and diafiltered with PBS, in about 2 h in aseptic conditions. The initial flux (45 L/m^2^ h) decreased of about 3-fold by the end of the process due to cake formation. Overall, the microfiltration and diafiltration steps yielded a 5.7-fold concentrated bacterial retentate, containing about 80 g of biomass. After addition of trehalose and sucrose the concentrated biomass was spray-dried resulting in a final recovery of about 84 g of powder. The recovered biomass showed a viability of 8.7 ± 3.1 × 10^10^ CFU/g of powder.

**FIGURE 3:**
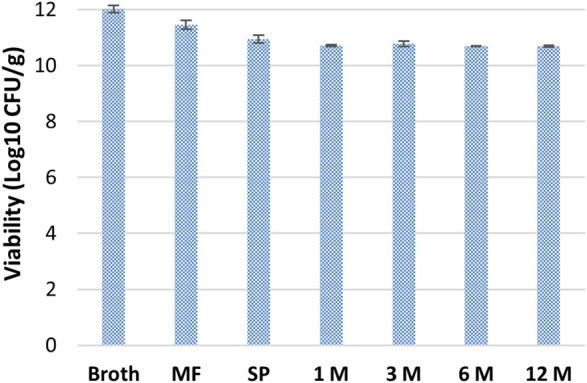
Stability of *B. lactis* HN019 during downstream treatment and storage at 4°C for 12 months. MF, Microfiltration; SP, Spray-drying.

After spray-drying, viability tests were carried out to verify stability for up to 12 months at 4°C. As demonstrated in Figure 3, the powder obtained suffered a 5% loss of viability during the concentration step on hollow fibers. A further 5% decrease was due to the spray-drying step. After that, the powder remained quite stable up to 12 months without additional reductions of viability.

### 3.4 EPS purification and analysis

The downstream protocol (Figure 1) was performed in parallel on 2 L of fresh medium (not inoculated) and on the same volume of broth recovered from batch fermentations to purify the EPS produced by *B. lactis* HN019. The medium containing yeast extract and soy peptone was digested with protease and ultrafiltered to reduce the presence of polysaccharidic contaminants. Results reported in Figure 1 indicate that protease treatment and first UF allowed to remove part of the contaminant polysaccharides (0.83 g) present in the complex nitrogen sources that represent 0.86% of the total medium components in weight (95.8 g). The analytical protocol applied to the medium was also useful to estimate the maximum potential contamination from medium polysaccharides in the partially purified EPS used for biological experiments, that was equal to 0.072 g, about 14% of the purified EPS.

During the ultrafiltration of the supernatant recovered from the broth the TMP stayed constant at 0.2 bar and on average the flux decreased from about 19 to 16 L/m^2^ h resulting in a process time of about 100 min.

**FIGURE 4:**
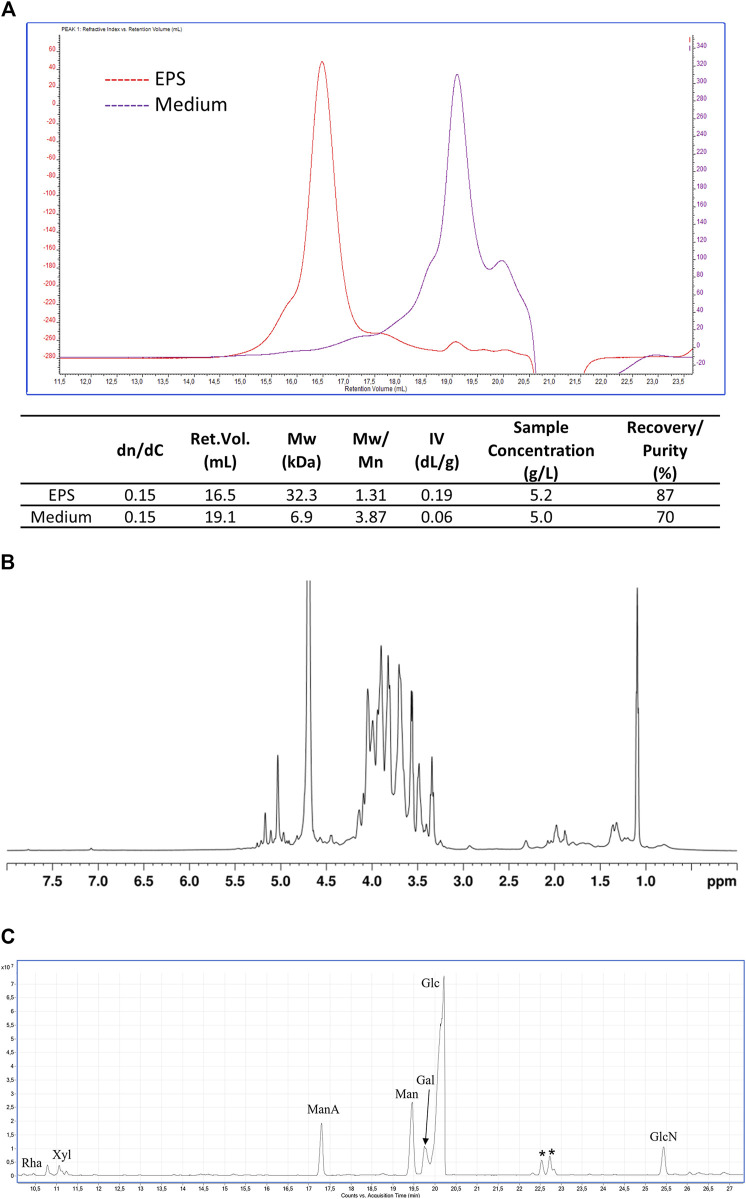
EPS characterization. **(A)** SEC-TDA analysis of the EPS produced by *B. lactis* HN019 and released in the fermentation broth and of the contaminants present in medium 4 isolated with the same downstream protocol. Red line, EPS; purple line, polysaccharides present in medium 4; **(B)** ^1^H-NMR spectrum (600 MHz, D2O, 25°C) of EPS produced by *B. lactis* HN019; **(C)** GC-MS chromatogram of AMGs from EPS produced by *B. lactis* HN019. Peaks marked with an asterisk refer to contaminants.

The solids recovered from the broth at the end of the purification procedure were equal to about 520 mg. The concentration of polysaccharides in the sample estimated by colorimetric assay (D’ambrosio et al., 2022) corresponded to about 8.2 mg/mL of glucose equivalents (457.6 mg in total) and presented a representativeness of about 88% excluding water. A similar result (87%) was obtained by analyzing the sample through SEC-TDA. The latter indicated the presence of a population of polymers with an average size of about 32.3 kDa and a polydispersity of 1.31 (Figure 4).

On the contrary the sample of oligo/polysaccharidic contaminants recovered applying the same purification protocol to the unspent medium used for fermentation experiments analysed by SEC-TDA showed a molecular population with an average size of about 6.9 kDa (Figure 4A). The most abundant fraction (67.6%) of the sample had a Mw of about 1.5 kDa, being thus oligosaccharides rather than polysaccharides.

The EPS sample was analyzed also by NMR, confirming the exclusive presence of polysaccharide material, as evidenced by the signals in the 4.9–5.3 ppm and 3.2–4.2 ppm regions of the ^1^H-NMR spectrum (Figure 4B), respectively attributable to the anomeric and the other ring hydrogen atoms of the monomeric constituents of polysaccharides. Conversely, no signals were detected in the aromatic region (7.0–8.0 ppm) of the spectrum, thus confirming the absence of protein/peptide and nucleic acid material in the sample. GC-MS analysis of monosaccharides derivatized as AMGs disclosed the presence of glucose (Glc) as the main sugar, and of galactose (Gal), mannose (Man), mannuronic acid (ManA), and glucosamine (GlcN). Finally, traces of rhamnose (Rha) and xylose (Xyl) were detected (Figure 4C). The relative ratios of the identified monosaccharides in the treated medium and in the purified EPSs are reported in Table 4.

**TABLE 4:** Relative ratio (molar %) of identified monosaccharides. *Rhamnose was below 0.1%.

	Rha	Xyl	ManA	Man	Gal	Glc	GlcN
Medium	-	-	-	43.2	33.6	21.2	2.0
EPS	n.d.*	0.5	1.1	2.1	1.1	94.5	0.7

### 3.5 TLR-4 and NF-kß expression by western blot assay

**FIGURE 5:**
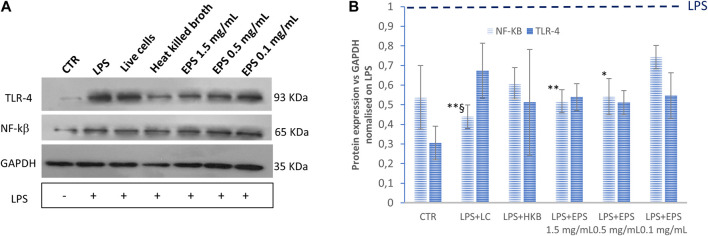
Expression levels of TLR-4 and NF-kß assessed by western blot assay. The values were first normalized with respect to the housekeeping protein GAPDH. The obtained values were further normalized on samples obtained by treating the cells with LPS only. The ratio sample/LPS is reported in the graph to show the relative change of expression in three biological replicates. HKB, heat killed broth; LC, live cells. Data were analysed by two-tailed non-homoscedastic Student’s t-test. *p < 0.05 vs. EPS 0.1 mg/mL; **p < 0.01 vs. EPS 0.1 mg/mL; §*p* < 0.05 vs. HKB.

The immunomodulatory effect of live *B. lactis*, heat-killed *B. lactis* broth, and EPS on LPS-induced inflammation in differentiated Caco-2 cells was determined by measuring the expression of TLR-4 and of the transcription factor NF-kß assessed by western blotting. All samples reduced the expression of TLR4; however, interestingly, when comparing samples with each other through two tailed non homoscedastic t-student tests, the highest doses of EPS (1.5 and 0.5 mg/mL) and the live cells most effectively affected TLR4 expression (Figure 5).

Accordingly, the effect of these treatments on the activation of the transcriptional nuclear factor NF-kß were also investigated. In this case, heat-killed *B. lactis* did not affect NF-kß expression significantly compared to cells treated with LPS only. Moreover, no significant difference could be found in response to live cells and to different EPS concentrations.

### 3.6 Assessment of tight junction protein zonulin (ZO-1) by immunofluorescence assay

To explicit the protective effect of live *B. lactis,* heat-killed *B. lactis* broth, and different concentrations of EPS (1.5, 0.5, and 0.1 mg/mL) on the intestinal barrier integrity, immunofluorescence assays were performed to observe the expression of tight junction protein ZO-1. Differentiated Caco-2 cells were insulted with 20 µg/mL LPS, and after 24 h the probiotics, and postbiotics, namely inactivated broth and different concentrations of EPS (1.5, 0.5, and 0.1 mg/mL), respectively, were administered.

**FIGURE 6:**
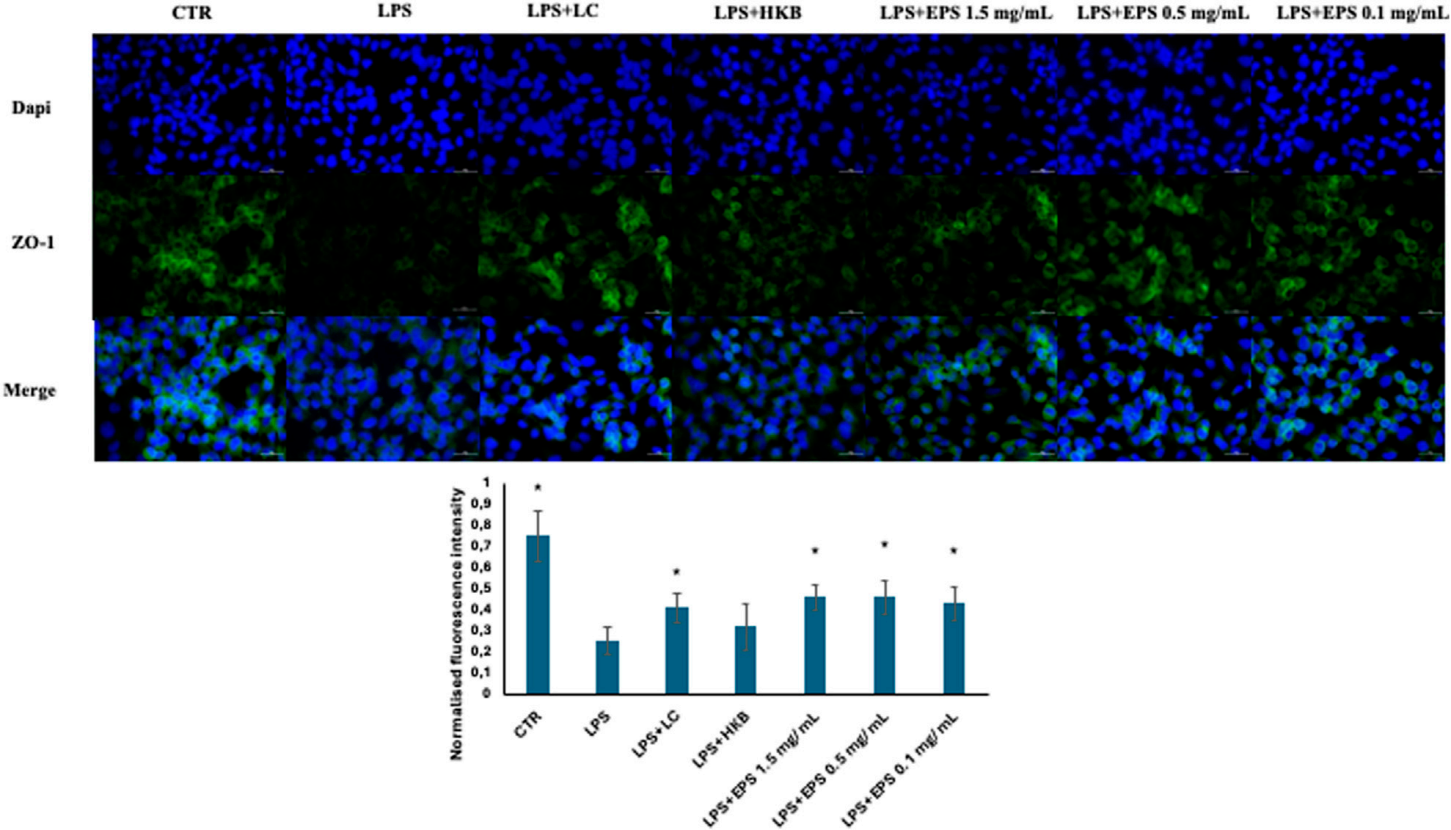
Immunofluorescence localization of ZO-1 in differentiated Caco-2 cells challenged with 20 µg/mL LPS, followed by the administration of live *B. lactis* HN019 (probiotics), Heat-inactivated broth of *B. lactis* HN019 and three different concentrations of EPSs (1.5, 0.5, and 0.1 mg/mL). Cells were fixed and stained with rabbit anti zonulin antibody (green stain), nuclei (dapi) in blue. Data were analyzed by two-tailed non homoscedastic Student’s t-test *Indicates *p* < 0.05 LPS vs. treatments.

As shown in Figure 6, the expression of tight junction protein-zonulin (ZO-1) was remarkably reduced in differentiated Caco-2 cells treated with the sole LPS. The reduced expression of ZO-1 protein was counteracted significantly by all samples, besides heat killed broth.

### 3.7 IL-6 secretion in inflamed Caco-2 cells induced by LPS and post treated with EPS

**FIGURE 7:**
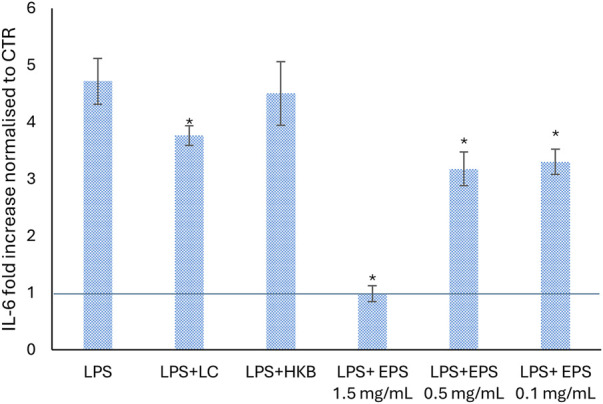
Effect of different concentrations of EPS (1.5, 0.5, and 0.1 mg/mL), live cells and heat killed broth of *B. lactis* HN019 on the level of IL-6 pro-inflammatory cytokine, secreted from differentiated Caco-2 cells after 24 h of LPS treatment. The change of IL-6 is presented as fold increase normalized in respect to untreated cells (CTR). Data were analyzed by two tailed homoscedastic Student’s t-test. *p < 0.05 indicates a statistically significance difference between treatments and cells treated with LPS only.

Since the pro-inflammatory cytokine IL-6 plays an important role in the cascade process initiated by the binding of the LPS to TLR-4, we examined the effects of different concentrations of EPS produced by *B. lactis* (1.5, 0.5, and 0.1 mg/mL) on the production of IL-6 in differentiated Caco-2 cells stimulated with LPS (Figure 7). Exposure to LPS led to a 5-folds significant increase in the production of IL-6 in respect to untreated cells (CTR). The following addition of EPS most effectively decreased the LPS-induced secretion of IL-6 from about 2 to 4 folds depending on the concentration used (Figure 7). A 1.4-folds reduced upregulation of LPS-induced IL-6 secretion was caused by live *B. lactic* cells, whereas the heat killed *B. lactis* broth did not affect IL-6 secretion.

## 4 Discussion

Bifidobacteria belong to one of the most abundant phyla (*Actinobacteria*) in the human intestine and can reach up to 90% of the bacterial population in breastfed infants. Therefore, specific live bifidobacteria, are commonly used as probiotics to maintain and especially restore a healthy intestinal state when the latter is threatened or lost due to diseases or other external causes.

In this respect, the development of simple and robust fermentation processes that deliver high yields of live probiotics, with reduced costs, are necessary. The rational design of a fermentation process is built on the evaluation of key physiological parameters, such as yield coefficients, glucose uptake rate, specific growth rate, and so on. In the last few years, several research groups characterized the growth and physiology of different strains of *Lactobacillaceae*, whereas very few data are available on bifidobacterial strains during fermentation (Desjardins et al., 1990; Van Der Meulen et al., 2006; Altieri et al., 2008). In the attempt to enhance biomass production, the evaluation of a suitable medium plays a central role in a fermentation process. In particular, LABs have limited biosynthetic abilities, and therefore vitamins and amino acids often need to be added to the medium. Often in fact MRS is used as medium for fermentation experiments, although the latter contains high amounts of animal derived complex nitrogen sources. Small scale bottle experiments were used to test the growth of *B. lactis* HN019 on four media differing for the amount of yeast extract and soy peptone, salts and other components (e.g., cysteine, maleic acid, tween) and all deprived of casein and meat extract contained in MRS. The highest concentration of biomass that reached 7.6 ± 0.2 OD_600_ was obtained on medium 4. The lack of MgSO_4_, that is involved in nutrient transport and enzymatic activity, did not have an impact on results, and also the different buffering systems used in the media resulted in the same final pH (4.0 ± 0.2). Dang and co-workers (Dang et al., 2021) demonstrated the importance of complex nitrogen sources for the growth of *B*. *animalis* subsp. *lactis* JNU306 using response surface methodology. Besides the latter, they tested three strains, namely, *B. longum* ATCC 15907, *B. bifidum* ATCC 35914 and *B. aminalis* subsp *lactis* BB12 on the optimized medium, demonstrating similar concentrations to those achieved on blood liver medium comprised between 7 and 9 log_10_ CFU/mL. However, here it seems that the total amount of complex nitrogen does not by itself justify the different biomass yields obtained in this work. In fact, the highest biomass concentration was found on the medium (4), with 35 g/L of total nitrogen sources, whereas decreasing the latter to 30 g/L (medium 2), reduced the final biomass titer by 50% (3.8 ± 0.1 OD_600_). On the other hand, medium 1 and 3, both containing only 20 g/L of complex nitrogen only showed a 15%–20% decreased biomass concentration. Differently from all others, in medium 2 the lower concentration of MnSO_4_ combined with the absence of sodium acetate, that acts as buffering agent and growth cofactor, might explain the lower biomass yield obtained. These data are in agreement with those reported by Galante and collaborators, optimizing medium design for the growth of *Latilactobacillus sakei* by one variable at time experiments (Galante et al., 2023). Ammonium citrate, was also present in all media tested besides medium 2; this salt is normally added to select for LAB strains and avoid growth of contaminants, and it was reported to promote growth of certain LAB (Waśko et al., 2010; Papizadeh et al., 2020). Additional experiments on medium 4 investigated the potential impact of higher concentrations of L-cysteine and maleic acid, and of the addition of lithium sulfate. L-cysteine, is a reducing agent that acts as an oxygen scavenger, keeping the redox potential low, and it was often reported to increase the viability of probiotic bacteria at low concentrations (Dave and Shah, 1997; Norouzbeigi et al., 2021). However, the growth of *Streptococcus thermophilus* was adversely affected by concentrations of cysteine as low as 50 mg/L and cellular damage was even found reaching 500 mg/L (Dave and Shah, 1997). Increasing L-cysteine from 0.5 normally present in M4 (control medium) to 2 g/L did not affect strain growth, whereas a fivefold higher titer of maleic acid, and the introduction of lithium sulfate resulted in a slight improvement of biomass production. However, the improvement achieved (6%) was not considered sufficient to switch medium. As expected, in comparison to MRS a lower concentration of biomass and of viable cells were obtained on M4, however this result is acceptable considering the overall impact of the replacement on safety and public concern issues.

Five different carbon sources were next evaluated (glucose, xylose, fructose, sucrose and lactose) to support the growth of *B. lactis* HN019 in medium 4. In particular, glucose and sucrose generated similar biomass concentrations, whereas lactose and especially fructose and xylose, yielded significantly lower values. This contradicts reports showing that some *B. lactis* strains prefer lactose to glucose and galactose (Van Der Meulen et al., 2004; Hsu et al., 2005). However, even closely related strains of *B. lactis* demonstrated different glucose utilization patterns/kinetics (Briczinski et al., 2008; 2009), including rapid growth on glucose as carbon source (Briczinski et al., 2009).

Batch experiments were performed to evaluate the key parameters for designing both the fed-batch and *ISPR* bioprocesses, such as maximal specific growth rate, yield coefficients and LA concentration. Results on the optimized medium in batch and FB experiments were quite encouraging considering previously described data with other *Bifidobacterial* strains (Dang et al., 2021), since on average 9.7 and 10.2 log_10_ CFU/mL were obtained, respectively. Since the accumulation of LA is responsible for the decrease of the cell specific growth rate as well as final biomass yield, these different fermentative strategies were tested to improve biomass production while also monitoring LA concentrations (and evaluating the possibility of obtaining two important biotechnological products in a single process).

As shown in Table 2 the ISPR experiments efficiently improved the final titer of viable cells; the latter, in fact, increased by about 230% and 98% in ISPR compared to that obtained exploiting batch and fed-batch fermentation processes, respectively. As expected, fed-batch experiments led to higher (220% compared to batch and 62% compared to ISPR) LA concentrations (82.9 ± 14.8 g/L). The significantly higher Y_x/s_ and lower Y_LA/s_ found in ISPR experiments confirm that lowering LA concentrations inside addresses the carbon flux towards biomass production, while a high concentration of LA during the growth resulted in positive feedback causing in the production of more LA. In fact, yield coefficients in ISPR and fed-batch showed opposite results. There is a lack of literature on similar fermentation strategies that improve growth of *B. lactis* HN019 at the bioreactor scale. *B. bifidum* reached a final viability of 2.2 × 10^10^ CFU/mL in a submerged membrane fermentation (Kwon et al., 2006). This result is quite similar to that obtained in the present work.

The purification of probiotic biomass with downstream approaches is another critical point (Dabous et al., 2023). Compared to traditional lyophilization processes, spray-drying is an economical process for the formulation of large quantities of viable microorganisms. It offers high production rates and low operating costs although, due to the high operating temperatures, it is usually less used for drying. Here, as demonstrated in Figure 3 trehalose and sucrose preserved biomass viability during the downstream process. In fact, a 9% decrease of the viability was only observed after microfiltration and spray-drying, whereas the biomass was stable up to 12 months during storage. Similar results were obtained by different groups that used maltodextrin or whey as stabilizers for biomass formulation (Shokri et al., 2015; Minj and Anand, 2022). Besides the application of live probiotics, recently the use of heat inactivated cells (postbiotics, often also indicated as parabiotics) and of metabolites produced from probiotic strains (postbiotics) are also emerging. In particular, since the exopolysaccharides produced by bifidobacterial could have a role in human health there is a renewed interest towards these polymers (Hidalgo-Cantabrana et al., 2016; Sabater et al., 2020; Oerlemans et al., 2021). Therefore, EPS production by *B. lactis* HN019 and its purification were studied to investigate potential biological effects.

Cell-free supernatants of lactobacilli and bifidobacteria have frequently been shown to possess antimicrobial properties (Fusco et al., 2023). However, to avoid potential interference from medium components, dedicated experiments were performed with a simplified medium. In particular, the medium was digested with protease and the ultrafiltration permeate was recovered and used for the growth of g of *B. lactis* HN019. The resulting medium still supported strain growth although the final concentration of biomass achieved during batch processes decreased by 30%.

These additional steps allowed a substantial removal of polysaccharides present in the complex nitrogen sources (yeast extract and soy peptone) that can co-precipitate with the target EPS during purification. The purified EPS analyzed by size exclusion chromatography triple detector array (SEC-TDA) showed a purity of about 87% (excluding water) and a molecular weight of about 32 kDa, whereas the contaminant polysaccharide purified with the same downstream treatment from the medium used for fermentation experiments, showed a quite different elution time (retention volume) and polydisperse population that barely overlapped with the EPS sample.

The EPS was further characterized by ^1^H-NMR and GC-MS. The ^1^H NMR analysis confirmed the exclusive presence of polysaccharide material, whereas the GC-MS chromatogram revealed the presence of mainly glucose, together with mannose, galactose, mannuronic acid, and glucosamine, some of which were also found in oligosaccharidic contaminants present in the medium.

Structurally and functionally different EPS have been found in bifidobacteria, as confirmed by the identification of highly diverse genes associated with EPS synthesis (Hidalgo-Cantabrana et al., 2014). Glucose, galactose and rhamnose are typically present in polysaccharides produced by bifidobacteria (e.g., *B. animalis*, *B. bifidum*, *B. breve*, *B. infantis* and *B. longum*). However, for example, the EPS from *B. longum* 35,624 was found to possess a particular repeating unit composed of galactose, glucose, galacturonic acid and 6-deoxytalose, a rather uncommon sugar (Altmann et al., 2016). In this work we found mannuronic acid in the partially purified EPS of *B. lactis* HN019. Mannuronic acid was not present in the sample of medium that underwent the same downstream procedure as that of the fermentation supernatant. This is a preliminary indication that the structure of the EPS from *B. lactis* HN019 may include mannuronic acid in its repetitive unit, therefore also potentially underlying diverse/additional biological activities.

In this study, the role of different concentrations of exopolysaccharides from *B. lactis* HN019 on the intestinal mucosal barrier and immune response, in LPS-induced inflammation in differentiated Caco-2 cells in an *in vitro* model, was also investigated, and their effects were compared with those of live and heat-killed *B. lactis* HN019 cells.

Intestinal tight junction destruction plays a major role in the development and progression of intestinal inflammation. ZO-1 is one of the main tight junction proteins involved in preserving this barrier, and its loss is frequently associated with infections and disease. In this study, the administration of different concentrations of EPS (1.5, 0.5, and 0.1 mg/mL) derived from *B. lactis* HN019, markedly restored the expression of ZO-1 after disruption due to exposure to LPS. These findings are consistent with results obtained by different research groups. Brdaric et al. investigated the effect of two concentrations of EPS (50 and 100 µg/mL) from *Lactiplantibacillus plantarum* BGAN8 against cadmium-induced toxicity in Caco-2 cells (Brdarić et al., 2021). They found that the mRNA fold expression of the tight junction protein ZO-1 was upregulated when cells were treated with the higher concentration of EPS, whereas both EPS concentrations restored the expression of the tight junction protein claudin (Brdarić et al., 2021). Moreover, Chen and his colleagues found that 2 mg/mL of EPS produced by *S. thermophilus* MN-BM-A01, upregulated the expression of three tight junction proteins ZO-1, occludin, and claudin after exposure to 10 µg/mL LPS in the Caco-2 monolayer (Chen et al., 2019). Liu et al. investigated the effect of EPS purified from *Lactobacillus helveticus* KLDS1.8701 on DSS (Dextran sodium sulfate)-induced colitis in mice (Liu et al., 2021). They found that the EPS has both anti-inflammatory and barrier stability effects, by alleviating the loss of ZO-1 and reducing the increase of inflammatory cytokines (IL-1ß, TNF-ɑ and IL-6) (Liu et al., 2021).

To further investigate the protective effect of EPS in an LPS-induced intestinal inflammation model, a western blot assay was conducted to explore the anti-inflammatory role of EPSs through the inhibition of the TLR-4/NF- kß pathway. TLR-4 is an upstream receptor and the main ligand for LPS. When LPS binds to TLR-4, this interaction may lead to an inflammatory response and to the production of inflammatory cytokines through the initiation of MAPK/NF-kß pathways (Zhao et al., 2022). Xu et al. observed that (100 µg/mL) EPS from *S. thermophilus* (EPS-3A) activated RAW264.7 macrophages through TLR-2 and TLR-4-mediated NF-κB and MAPK pathways (Xu et al., 2022). In a recent study by Li et al., the authors found that various concentrations of EPS (0.5, 1 and 2 mg/mL) of *L*. *rhamnosus* GG ameliorated *Salmonella typhymurium*-induced inflammation of mouse intestinal cells, by inhibiting the activation of the TLR-4/NF-kß pathway (Li et al., 2023). In another study conducted by Kwon and his colleagues (2020), the inhibitory effect of (100 and 200 µg/mL) EPS from *L. plantarum* L-14 in response to LPS was investigated. This EPS, mainly composed of glucose, inhibited the production of the pro-inflammatory mediator NF-kß through the suppression of TLR-4 in mouse macrophages (RAW 264.7) cells (Kwon et al., 2020). In our previous research, both live *Levilactobacillus brevis* SP-48 cells and bioactive compounds purified from the fermentation broth, exerted an immunomodulatory effect by downregulating the expression of TLR-4 and NF- kß in differentiated Caco-2 cells after exposure to LPS (Cimini et al., 2022).

In this study, cells treated with live *B. lactis*, inanimate *B. lactis* broth, as well as with different concentrations of EPS (1.5, 0.5, and 0.1 mg/mL) reduced the expression of NF-kß, after LPS induced overexpression, as indicated by western blotting experiments results. Interestingly, although all samples affected NF-kß expression, results obtained from treatments with live cells as well as with higher EPS concentrations (1.5 and 0.5 mg/mL) were significantly different, indicating that lower EPS concentrations and heat inactivated cells were less effective. TLR-4 expression was also significantly lowered, following LPS treatment, from all samples besides from postbiotics in the form of heat killed broth. This might be due to high sample variability or to the involvement of other toll like receptors (e.g., TLR-2).

Moreover, EPS and live probiotic cells increased the expression of the tight junction protein-zonulin in LPS-treated Caco-2 cell, potentially improving intestinal barrier integrity. In this case, no significant difference was detected between the different concentrations of EPS.

Our results also showed the ability of different concentrations of EPS to downregulate the secretion of IL-6 induced by LPS treatment for 24 h (Castro-Herrera et al., 2020; Noh et al., 2022). All concentrations of EPS (1.5, 0.5, and 0.1 mg/mL) significantly attenuated the secretion of IL-6 after LPS treatment, but only the highest one restored the condition observed in untreated cells.

As previous reports have suggested (some but not all) that the anti-inflammatory effect of probiotics can be exerted by both live and heat-inactivated probiotics, we compared the effects of live and heat-inactivated *B. lactis* on IL-6 production in Caco-2 cells. A reduction of IL-6 was caused by live cells, whereas no effect was found following treatment with heat inactivated *B. lactis* HN019. Similar findings were described by Reilly and colleagues, (Reilly et al., 2007), in fact heat-inactivated *Lacticaseibacillus paracasei* affected IL-6 production in Caco-2 cells stimulated by IL-1ß (Reilly et al., 2007). On the other hand, other studies in which the LPS was administered either just before or simultaneously with LPS the anti-inflammatory efficiency of heat-killed probiotics was demonstrated (Castro-Herrera et al., 2020; Ladda et al., 2023; Xie et al., 2023). This divergence might be explained by differences in the time of treatment that can occur either after, before, or simultaneously with the inflammatory stimulus. For instance, in the model used in the present work, Caco-2 cells were stimulated with LPS for 24 h prior to HK cells administration, a lot earlier and a lot longer compared to other studies (Castro-Herrera et al., 2020; Ladda et al., 2023; Xie et al., 2023).

## 5 Conclusion

Overall, this study demonstrates the possibility to improve the titers of live *B. lactis* HN019 cells by using efficient fermentation strategies on a medium that is deprived of animal derived components, making the application of this probiotic strain safer and open to a larger audience of customers (e.g., vegetarian, vegans). Moreover, it demonstrates that medium pre-treatment and a simple downstream procedure enriched the representativity of the exopolysaccharide recovered (87%), the composition of which was also investigated in the study revealing the presence of mannuronic acid. Finally, besides live cells also the EPS purified samples, especially at higher concentrations, demonstrated an anti-inflammatory effect as well as the ability to promote intestinal barrier integrity, and can therefore be considered as potential effective adjuvants in gut disorders. Heat inactivated broth only affected the expression of NF-kß, indicating a lower potential compared to the other samples, in the tested conditions.

The use of heat inactivated fermentation broth or partially purified bioactive molecules simplifies the process since the hurdle of maintaining high viability levels during probiotic preparation can be neglected. In this respect data provided in this work are relevant to drive further biotechnological investigations.

## Data Availability

The raw data supporting the conclusion of this article will be made available by the authors, without undue reservation.
