# Supplementary material for: Bifidobacterium animalis subsp. lactis HN019 live probiotics and postbiotics: production strategies and bioactivity evaluation for potential therapeutic properties
Source: Front Bioeng Biotechnol. 2024 Jul 9;12:1379574. doi: 10.3389/fbioe.2024.1379574 (PMC11270027; doi:10.3389/fbioe.2024.1379574)

frontiers in Bioengineering and Biotechnology

"*Bifidobacterium animalis* subsp. *lactis* HN019 live probiotics and postbiotics: production strategies and bioactivity evaluation for potential therapeutic properties".

Trial 1 TLR-4

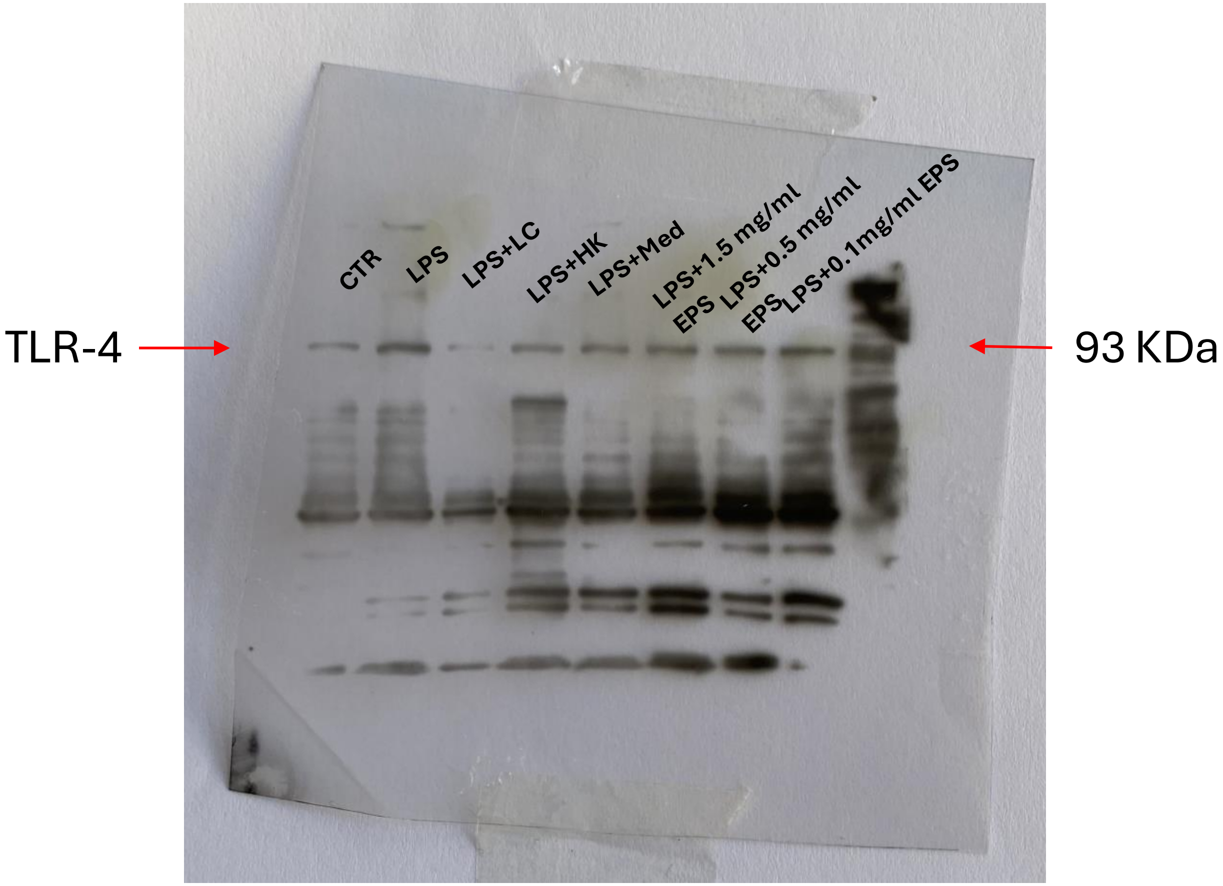
CTR
LPS
LPS+LC
LPS+HK
LPS+Med
LPS+1.5 mg/ml EPS
LPS+0.5 mg/ml EPS
LPS+0.1mg/ml EPS
TLR-4
93 KDa

Trial 1 NF-KB

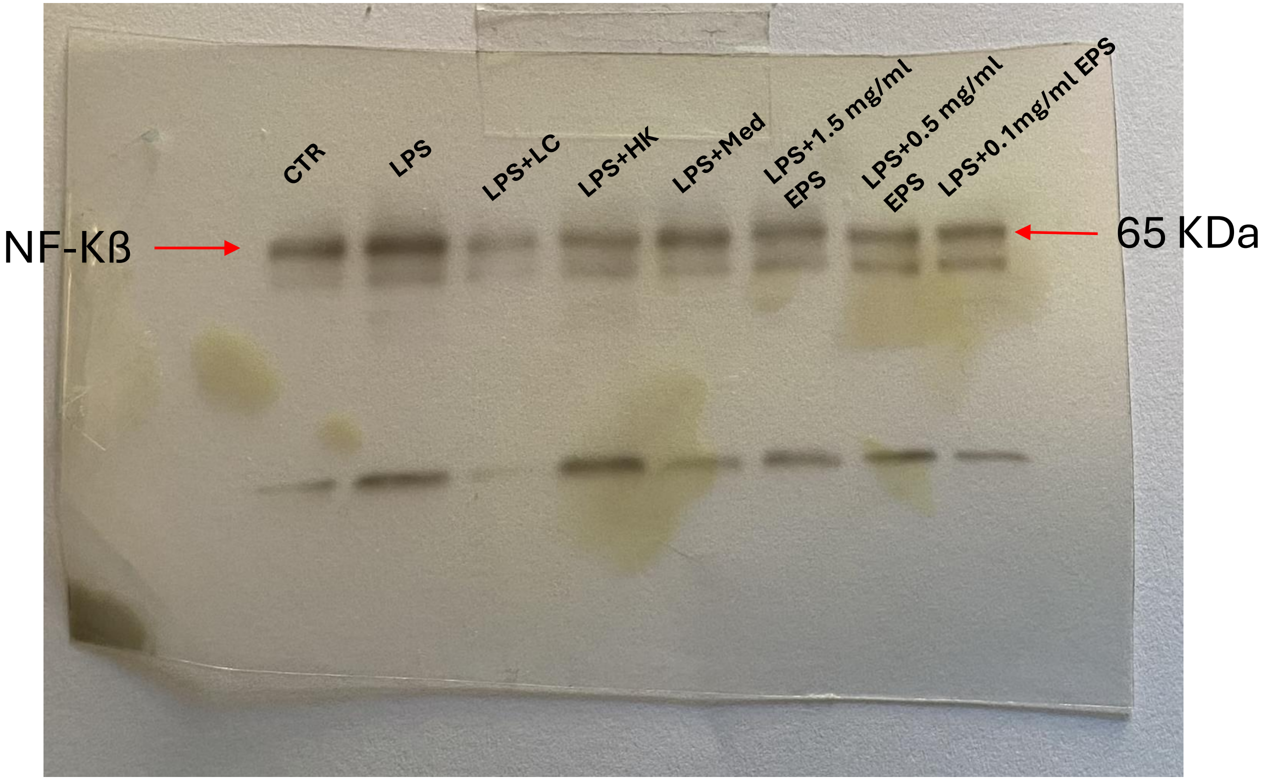
CTR
LPS
LPS+LC
LPS+HK
LPS+Med
LPS+1.5 mg/ml EPS
LPS+0.5 mg/ml EPS
LPS+0.1mg/ml EPS
NF-Kβ
65 KDa

Trial 1 GAPDH

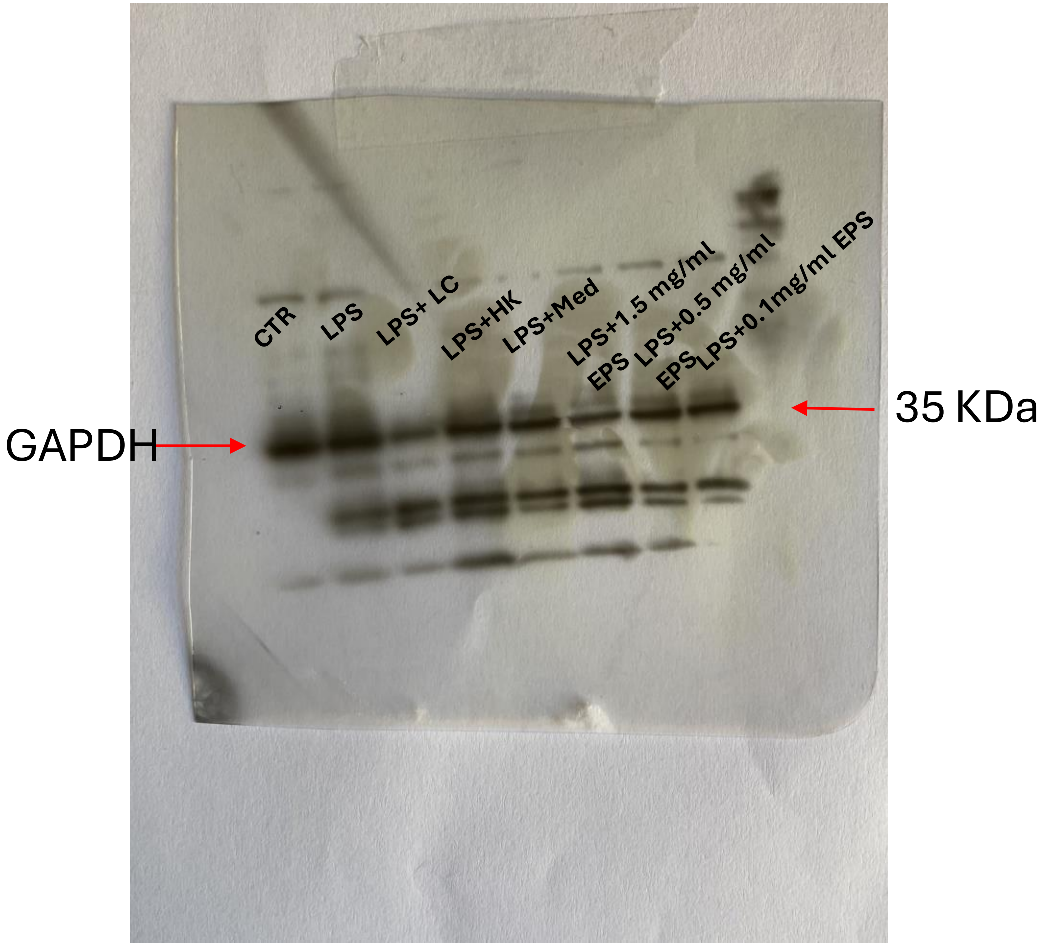

Trial 2 TLR-4

TLR-4
NF-KB old signal

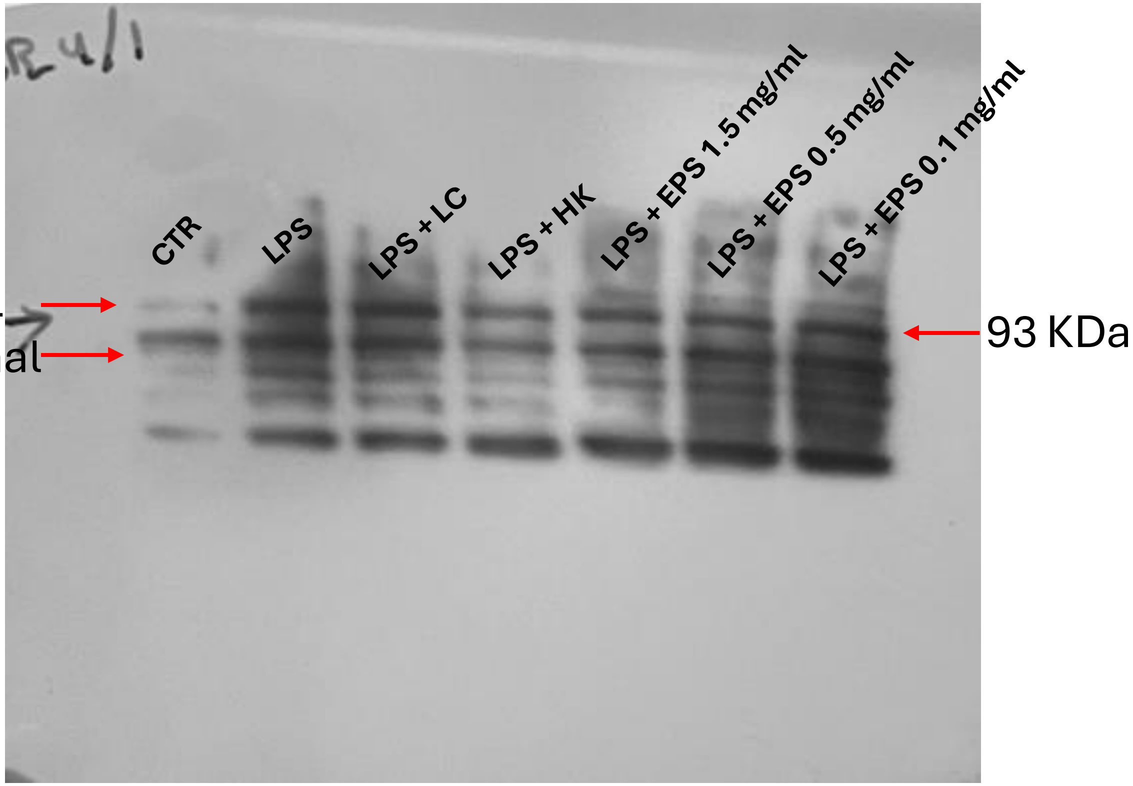

93 KDa

After processing

TLR-4

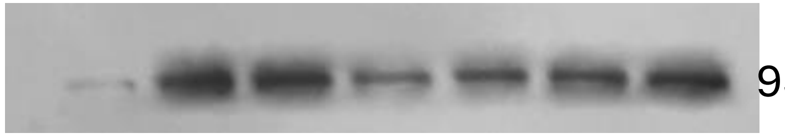

93 KDa

Trial 2 NF-KB

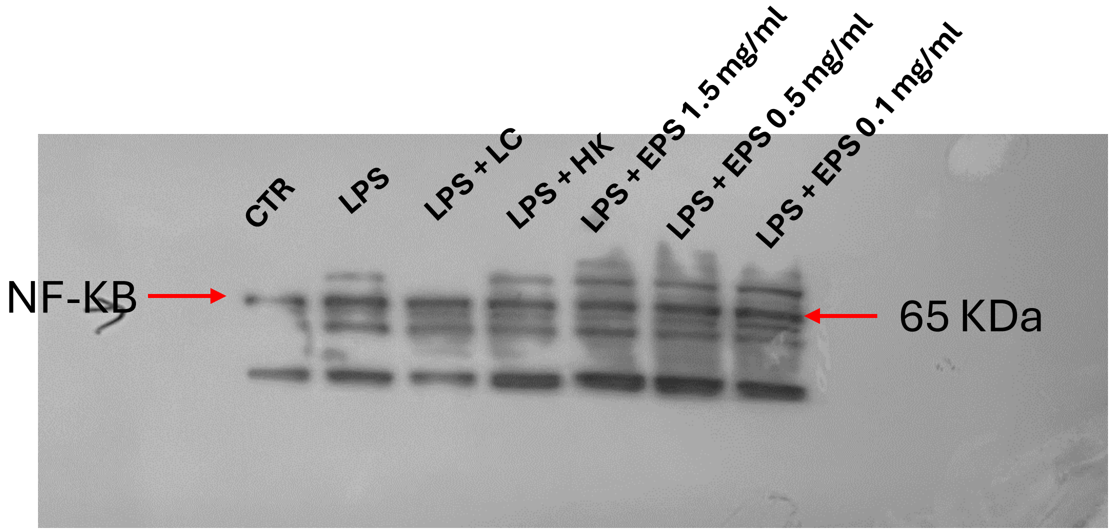

After processing

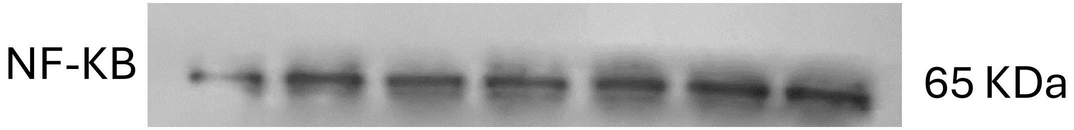

Trial 2 GAPDH

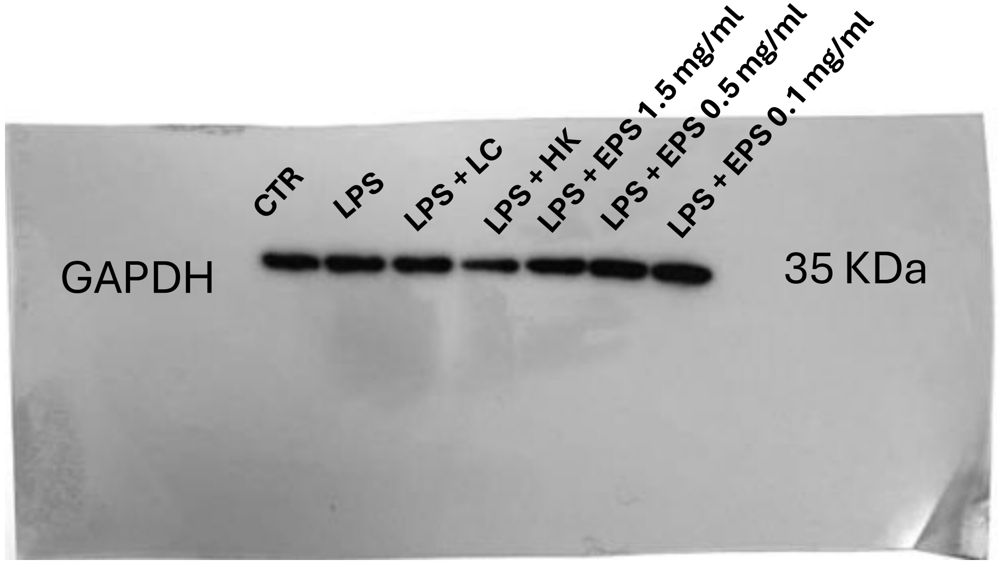

Trial 3- GAPDH

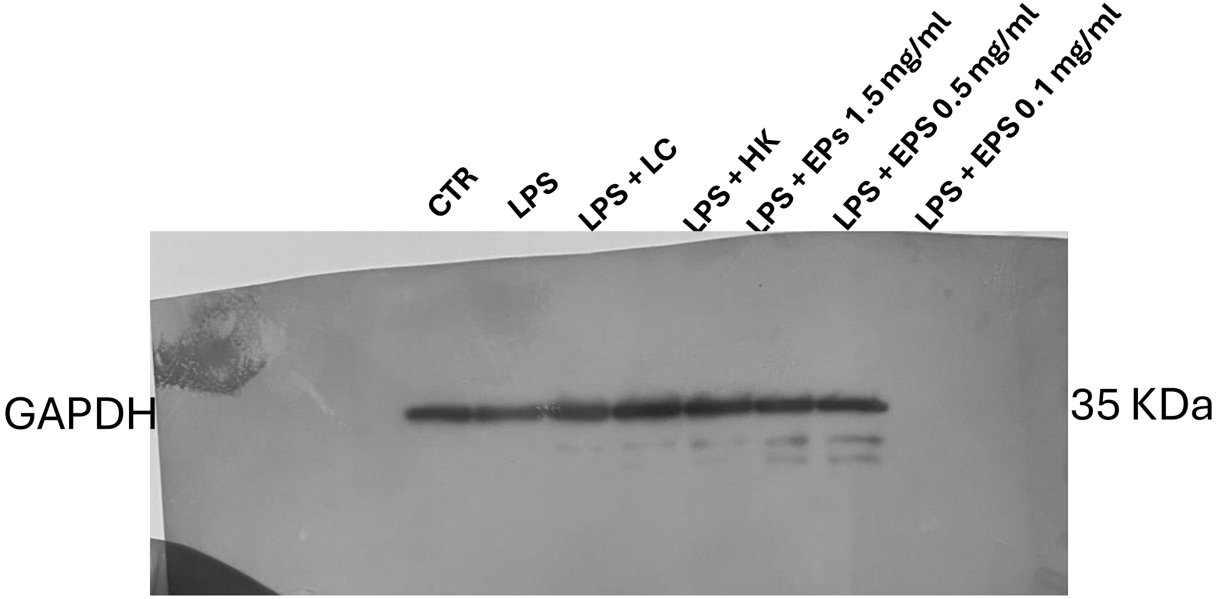

Trial 3 NF-kB

After processing → NF-KB

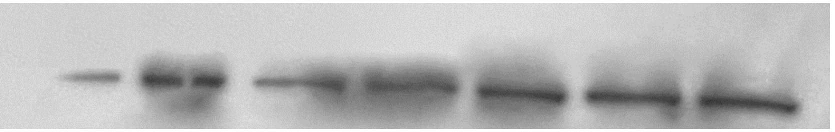

65 KDa

NF-KB →

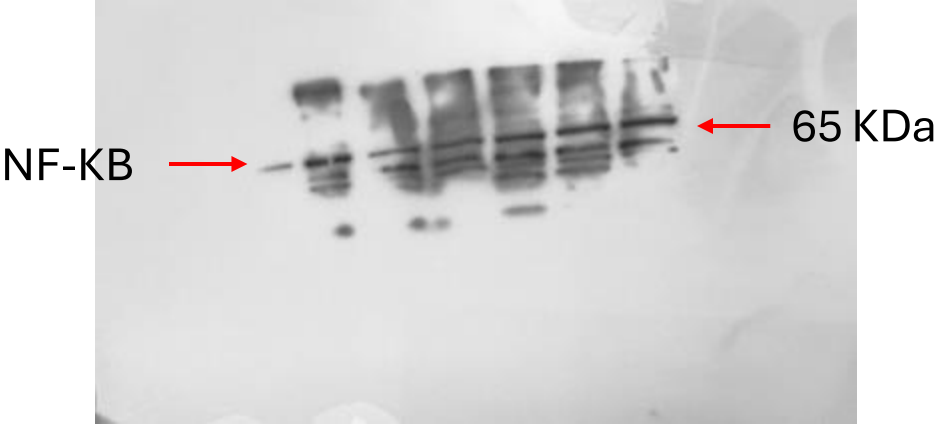

← 65 KDa

Trial 3 TLR-4

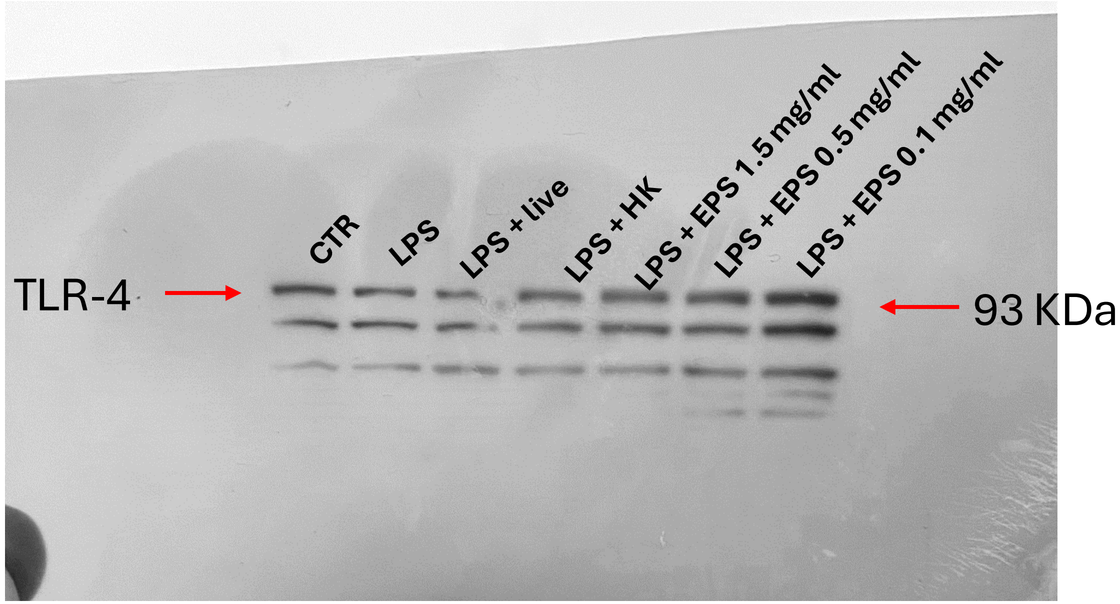

CTR
LPS
LPS + live
LPS + HK
LPS + EPS 1.5 mg/ml
LPS + EPS 0.5 mg/ml
LPS + EPS 0.1 mg/ml
TLR-4
93 KDa

Figure from trial 2 presented in the manuscript

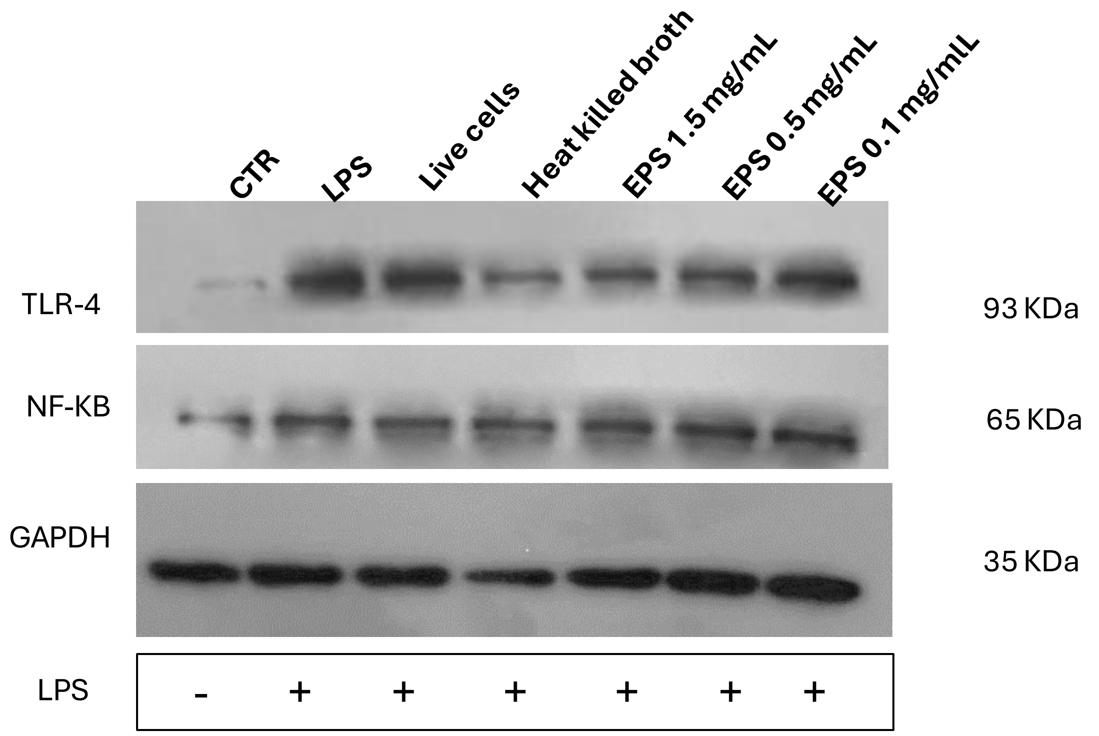

Supplement: Supplementary file 1 [file Image1.pdf]
